# Expired Patents

**Published:** 1907-10

**Authors:** 


EXPIRED PATENTS.

August 26, 1907.

434952. Dental Engine Hand Piece, C. M. Richmond, New York, N. Y.
435138. Angle Attachment for Dental Engines, F. Fleury and A. G. Goodman, Chicago, Ill.
435228. Rubber Dam Clamp, J. W. Ivory, Philadelphia, Pa.

September 9, 1907.

436009. Locking Device for the Flasks of Dental Vulcanizers, A. H. Stoddard, Boston, Mass.
436210. Dental Forceps, B. E. Burger, Merrill, Wis.

September 30, 1907.

437319. Dental Engine, E. Thompson, San Francisco, Cal.

The above lists of patents are furnished by Davis & Davis, Solicitors of American and Foreign Patents. Washington, D. C., and St. Paul Building, New York City.

